# Changes in pulmonary function and feasibility of portable continuous laryngoscopy during maximal uphill running

**DOI:** 10.1136/bmjsem-2020-000815

**Published:** 2020-08-24

**Authors:** Mette Engan, Ida Jansrud Hammer, Trine Stensrud, Hilde Gundersen, Elisabeth Edvardsen, Hege Havstad Clemm

**Affiliations:** 1 Department of Pediatric and Adolescent Medicine, Haukeland University Hospital, Bergen, Norway; 2 Institute of Clinical Science, University of Bergen, Bergen, Norway; 3 Institute of Sports Medicine, The Norwegian School of Sport Sciences, Oslo, Norway; 4 Department of Sport, Food and Natural Sciences, Western Norway University of Applied Sciences, Bergen, Norway; 5 Institute of Physical Performance, The Norwegian School of Sport Sciences, Oslo, Norway

**Keywords:** Exercise testing, Feasibility, Lungs, Outdoor

## Abstract

**Objective:**

To evaluate changes in pulmonary function and feasibility of portable continuous laryngoscopy during maximal uphill running.

**Methods:**

Healthy volunteers participated in an uphill race. Forced expiratory volume in 1 s (FEV_1_) and forced vital capacity (FVC) were obtained before and 5 and 10 min after finishing the race. Capillary blood lactate concentration ([BLa^-^]) and Borg score for perceived exertion were registered immediately after the race. One participant wore a portable video-laryngoscope during the race, and the video was assessed for technical performance.

**Results:**

Twenty adult subjects participated with a mean (SD) age of 40.2 (9.7) years. Mean (SD) race duration and post-exercise [BLa^-^] was 13.9 (2.3) min and 10.7 (2.1) mmol/L, respectively, and the median (range) Borg score for perceived exertion was 9 (5–10).

Mean percentage change (95% CI) 5 and 10 min post-exercise in FEV_1_ were 6.9 (3.7 to 10.2) % and 5.9 (2.7 to 9.0) %, respectively, and in FVC 5.2 (2.3 to 8.1) % and 4.7 (1.6 to 7.9) %, respectively. The recorded video of the larynx was of good quality.

**Conclusions:**

Maximal aerobic field exercise induced bronchodilatation in the majority of the healthy non-asthmatic participants. It is feasible to perform continuous video-laryngoscopy during heavy uphill exercise.

## INTRODUCTION

Airway calibre is an important determinant of airflow and is dynamically regulated by both neural and humoral factors.^1^ During exercise, the lower intrathoracic airway calibre may change because of alterations in the parasympathetic/sympathetic balance, increase in circulating catecholamines and release of prostaglandins by mast cells and airway epithelial cells.^1^ While much is understood about control of airway calibre at rest, less is known about dynamics and control of airway calibre during exercise.^2^

In non-asthmatic subjects, studies suggest that complete withdrawal of parasympathetic control of airway tone occurs during moderate exercise, resulting in either a mild or no reduction of airway resistance.^1–6^ Reports on airway response to exercise are mostly done indoor in laboratory settings, conditions not necessarily able to induce the similar change in airway calibre that may occur during or after outdoor sport-specific exercise.^7 8^ To our knowledge, airway response to maximal field exercise challenges in non-asthmatic subjects has not been explored. Outdoor exercise challenges on healthy non-asthmatic subjects would give valuable information on normal physiological changes in the airways.

The possibility that respiratory symptoms during exercise are caused by dysfunction in the larynx has been increasingly recognised in the last decade.^9^ Exercise-induced laryngeal obstruction (EILO) is a condition where closure of the laryngeal inlet impacts breathing solely during strenuous exertion but appears to be normal at rest. To determine if EILO is present, a continuous laryngoscopy during exercise (CLE) test is used and considered the ‘gold standard’.^10^

CLE tests are mainly done indoor on a treadmill or stationary bicycle. However, there are some reports on sports-specific CLE tests performed on a few individuals during indoor rowing,^11^ swimming in resistance pool^12^ and during various forms of exercise, that is, running at moderate pace, cycling, stairs climbing and shuttle walking.^13^ These tests were successfully completed with acceptable user comfort, no adverse events and satisfactory image quality of the larynx.^11–13^ However, there has not yet been done an outdoor CLE test during strenuous exercise, neither during a competition.

In order to study pulmonary response to maximal aerobic field exercise, our primary aim was to determine the change in forced expiratory volume in 1 s (FEV_1_) and forced vital capacity (FVC) following a steep uphill race in non-asthmatic subjects. Secondary, we aimed to determine the feasibility of performing continuous video-laryngoscopy during exercise (CLE) during steep uphill running to explore the diagnostic opportunities for EILO.

## METHODS

### Participants and study design

This study was a cross-sectional and feasibility study. Twenty volunteers without current asthma attending the annual Norwegian Sports Medicine Conference in 2018 were recruited to participate in an outdoor uphill race to evaluate the change in pulmonary function immediately after a maximal aerobic exercise challenge. Ethical approval was granted by the regional ethics committee, and informed written consent was obtained from all participants.

The day before the race, all participants completed the *Modified AQUA2008—Questionnaire for assessment of asthma, allergy and other respiratory disorders for athletes participating in the summer Olympic games in Beijing, August 2008* ^14^ with additional questions on inspiratory breathing symptoms. *Previous asthma* was defined as previous use of asthma medication in childhood/youth. *Atopic disease* was defined as asthma, allergic rhinitis or conjunctivitis, allergic urticaria, atopic eczema, food allergy or drug allergy ever diagnosed by a physician. Only subjects without current asthma were included in the study.

The race was in a steep trail with approximately 900 stair steps up. The length of the race was 834 m, with an increase of 301 altitude meters and an average incline of 36%. The participants were asked to complete the distance as fast as possible and had a short warm-up before start. Each individual began running at 30 s interval.

### Pulmonary function

Pulmonary function was measured as maximal expiratory flow volume curves. Baseline and 5 and 10 min post-exercise FVC, FEV_1_, forced expiratory flow at 50% of exhaled FVC (FEF_50_) and FEV_1_/FVC were obtained according to standard quality criteria.^15^ Two spirometry devices were used in the study: (1) the *Vyntus Spiro* (Vyaire GmbH, Höchberg, Germany) with bacterial filter from Medical Respiratory Devices S.L. (Madrid, Spain) and (2) *Smart pft USB* (Medical Equipment Europe GmbH, Hammelburg, Germany) with bacterial filter *Pulmosafe V3/2* (LemonMedical GmbH, Hammelburg, Germany). The same spirometry device was used pre-exercise and post-exercise for each individual. The z-values for FVC and FEV_1_ were calculated by the Global Lung Function Initiative online spirometry calculator.^16^

### Blood lactate concentration and Borg score

Elevation of capillary blood lactate concentration ([BLa^-^]) (Lactate Scout+, EKF Diagnostic GmbH, Ebendorfer Chaussee 3, 39 179 Barleben, Germany) and perceived exertion registered with Borg score (0–10)^17^ were used to describe the participants exercise intensity. A [BLa^-^] of ≥8 mmol/L is often considered to indicate that maximal oxygen consumption is reached.^18^

### Continuous laryngoscopy during exercise

**Figure 1:**
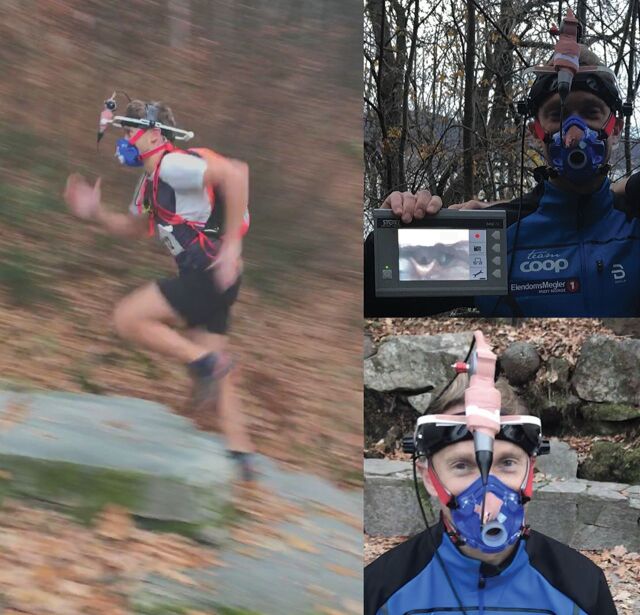
The participant at rest and running with the portable video-laryngoscope attached. The monitor shows the video-recording of the laryngeal area (upper right image).

One volunteering participant had a portable video-laryngoscope attached during the race (CMOS Video-Rhino-Laryngoskope and 8402ZX monitor, Karl Storz GmbH & Co, Tuttlingen, Germany). In the starting-area of the race, personnel familiar with the equipment inserted the video-laryngoscope in one participant’s pharyngeal space after application of topical lidocaine in one nostril. The handle of the laryngoscope was attached to a special headgear and a customised Rudolph-mask held the laryngoscope in position. The monitor was placed in a small backpack carried by the participant (figure 1). The recorded video of the larynx from the continuous video-laryngoscopy was assessed for technical quality by a trained physician (HHC) familiar with indoor CLE testing and the EILO diagnosis.

### Data analysis

Normally distributed data were presented as means with SD and skewed data as median with ranges. Intraindividual changes in spirometry values were calculated using dependent Student’s t-test and reported as percentage change and mean change with 95% CI.

To detect a mean difference in FEV_1_ of 5% with an assumed SD of 6% in a two-tailed paired t-test with an alpha value at 0.05 and a power of 90%, we needed 19 participants to complete the test.^19^ A p value ≤0.05 was considered statistically significant. Analyses were performed using IBM SPSS statistics version 24.

The feasibility of CLE testing during uphill running was determined by the time taken to mount the CLE equipment, user comfort and the recorded video’s ability to visualise the larynx during the race enabling EILO assessment.

## RESULTS

Characteristics of the participants and race results are presented in table 1.

**Table 1:** Background characteristics and race results of 20 healthy adults completing a short maximal aerobic field exercise

Variables, units			Range
Male gender, n (%)	17	(85)	
Age, years (SD)	40.2	(9.7)	
BMI, kg/m^2^ (SD)	23.8	(2.7)	
Cigarette smoking, n (%)	0	(0)	
Moist snuff, n (%)	2	(10)	
Asthma previous,* n (%)	4	(20)	
Atopic disease,† n (%)	6	(30)	
Exercise >3 times/week, n (%)	10	(50)	
Exercise daily, n (%)	7	(35)	
Ever shortness of breath/coughing during/after exercise, n (%)	5	(25)	
Race time, min, mean (SD)	13.9	(2.3)	10.4–19.6
Lactate, mmol/L, mean (SD)	10.7	(2.1)	4.7–14.0
Borg score perceived exertion (0–10),‡ median	9		5–10
Inspiratory stridor, n (%)	1	(5)	

*Asthma previous defined as previous use of asthma medication in childhood/youth.

†*Atopic disease* defined as asthma, allergic rhinitis or conjunctivitis, allergic urticaria, atopic eczema, food allergy or drug allergy ever diagnosed by a physician.

‡Borg score grades perceived exertion from ‘0=nothing at all, 0.5=extremely weak, 1=very weak, 2=weak, 3=moderate, 4=somewhat strong, 5=strong, 7=very strong, 9=very, very strong, 10=extremely strong’.^17^

Spirometry values and changes in FEV_1_ and FVC from baseline to 5 and 10 min post-exercise are presented in table 2.

**Table 2:** Spirometry values obtained from 20 healthy participants before and after completing a short maximal aerobic field exercise

	Pre-exercise		5 min post-exercise		10 min post-exercise		% change at 5 min		Change at 5 min			% change at 10 min			Change at 10 min			
Spirometry values, units	Mean	SD	Mean	SD	Mean	SD	Mean diff %	95% CI %	Mean diff		95% CI	P value*	Mean diff %	95% CI %	Mean diff		95% CI	P value*
FEV_1_, L	4.33	0.58	4.64	0.78	4.59	0.75	6.9%	3.7% to 10.2%	0.31		0.15 to 0.47	0.001	5.9%	2.7% to 9.0%	0.26		0.12 to 0.41	0.001
FEV_1_, z-value	0.23	0.76	0.84	1.19	0.75	1.09			0.61		0.30 to 0.92	0.001			0.51		0.23 to 0.80	0.001
FVC, L	5.41	0.88	5.70	1.01	5.67	1.03	5.2%	2.3% to 8.1%	0.29		0.12 to 0.45	0.002	4.7%	1.6% to 7.9%	0.26		0.08 to 0.34	0.006
FVC, z-value	0.23	0.62	0.67	0.93	0.63	0.91			0.44		0.20 to 0.68	0.001			0.40		0.14 to 0.66	0.005
FEF_50_, L/s	5.29	1.38	5.68	1.41	5.48	1.34	8.2%	2.0% to 14.4%	0.39		0.12 to 0.67	0.007	4.5%	0.1% to 8.9%	0.19		−0.05 to 0.44	0.113
FEV_1_/FVC, units	0.81	0.06	0.82	0.07	0.81	0.06			0.14		0.00 to 0.03	0.027			0.01		0.00 to 0.02	0.120

*Dependent sample t-test.

Change in %: (Post-exercise value−pre-exercise value)/pre-exercise value×100.

diff, difference; FEF_50_, forced expiratory flow at 50% of exhaled FVC; FEV1, forced expiratory volume in 1 s; FVC, forced vital capacity.

Nine participants (45%) had an increase in FEV_1_ of ≥8%, and among them, five participants (25%) had an increase of ≥10% after the race. None of the participants had a decrease in FEV_1_ of more than 8%. Six participants (30%) had an increase in FVC of ≥8%. The FEV_1_/FVC ratio increased significantly only 5 min post-exercise.

The portable video-laryngoscope was mounted in less than 10 min and was well tolerated by the participant who was able to perform at high intensity with a [BLa^-^] of 12.5 mmol/L and a Borg score for perceived exertion of 9 (very, very strong) after completing the race. The CLE video was of good quality during the field race, visualising the laryngeal movements.

## DISCUSSION

To our knowledge, this is the first study to investigate changes in pulmonary function in healthy adult individuals after a short maximal aerobic field exercise. The mean increase in FEV_1_ 5 min post-exercise was about 7% in middle-aged non-asthmatic individuals. A quarter of the participants had an increase in FEV_1_ of ≥10% and 30% of the participants had an increase in FVC of ≥8% post-exercise. A significant increase in mean FEV_1_/FVC ratio at 5 min post-exercise was observed, implying airway dilatation.

Diverging to our results, previous studies on non-asthmatic individuals in the laboratory have found that exercise induces either no change or a small decrease in airway resistance.^1–6^ However, in studies where the airways are pharmacological pre-constricted, exercise is shown to be a strong bronchodilator in non-asthmatic individuals.^2 20 21^ Interestingly, similar bronchodilatation has also been reported in healthy subjects with pre-constricted airways after isocapnic hypopnea, suggesting that increased ventilation itself and not exercise induces bronchodilation.^22^ In our study on non-asthmatic individuals, we do not presume that the participants had an increased resting vagal tone at pre-exercise spirometry, and this could therefore not be the explanation to our findings.

Airway calibre in healthy persons shows diurnal variation, and FEV_1_ is shown to be lower in the morning and about 4% higher in the afternoon.^23^ In this study, the pre-exercise spirometry was performed in the afternoon and the post-exercise spirometry was performed in the morning at about 09:00. Thus, the average increase found in FEV_1_ might be underestimated.

Studies on lung function after exercise of various intensity and duration report a decline in FVC, possibly explained by respiratory muscle fatigue.^24^ We found that mean FVC increased by approximately 5% after the race compared to pre-exercise values (table 2). We suggest that these findings represent recruitment of non-ventilated lung areas and dilatation of the airways induced by the maximal aerobic exercise challenge. However, our findings need to be confirmed in future studies.

A limitation of our results on changes in pulmonary function is the small sample size with an uneven gender distribution. The recruitment was based on volunteerism and generalisation of the results should be made with caution.

The one participant wearing the laryngoscope reported not to be hampered by the equipment and performed close to his maximal effort. The recorded video was of good quality enabling clear visualisation of the larynx throughout the race. This study shows that it is possible to do a personalised CLE test to characterise laryngeal movement during outdoor sport-specific exercise like uphill running.

EILO is an important differential diagnosis when assessing respiratory symptoms during exercise with implication for treatment.^25^ There are a few feasibility reports on sports-specific CLE tests performed on a few individuals.^11–13^ Our report is the first CLE test done outdoor during maximal effort and competition. However, the result is limited by including only one participant; thus, future studies should aim to determine the validity and reliability of ambulant CLE testing to improve diagnostic opportunities.^13^

## CONCLUSIONS

We found that maximal aerobic field exercise induced bronchodilatation in the majority of the healthy non-asthmatic participants. Similar field studies are lacking, and our results need to be confirmed in future studies. Our study also showed the feasibility of using a portable video-laryngoscope during heavy uphill running, which is a step forward to do an advanced assessment of respiratory problems during ‘real-life’ exercise.

**What are the new findings?**

- Maximal uphill running induced bronchodilatation in non-asthmatic participants.
- It is feasible to use a portable video-laryngoscope during heavy uphill running.
- Future studies should aim to determine validity and reliability of field CLE testing.
